# Validation of a 1:8 Scale Measurement Stand for Testing Airborne Sound Insulation

**DOI:** 10.3390/s21196663

**Published:** 2021-10-07

**Authors:** Agata Szeląg, Katarzyna Baruch-Mazur, Krzysztof Brawata, Bartosz Przysucha, Dominik Mleczko

**Affiliations:** 1 Wind Engineering Laboratory, Faculty of Civil Engineering, Tadeusz Kościuszko Cracow University of Technology, ul. Warszawska 24, 31-155 Kraków, Poland; 2 Gorycki & Sznyterman Sp. z o.o., ul. T. Chałubińskiego 53, 30-698 Kraków, Poland; katarzyna.baruch@fpgs.pl (K.B.-M.); krzysztof.brawata@fpgs.pl (K.B.); 3 Department of Quantitative Methods in Management, Faculty of Management, Lublin University of Technology, ul. Nadbystrzycka 38 D, 20-618 Lublin, Poland; b.przysucha@pollub.pl; 4 Department of Mechanics and Vibroacoustics, Faculty of Mechanical Engineering and Robotics, AGH University of Science and Technology, al. Adama Mickiewicza 30, 30-059 Kraków, Poland; dmleczko@agh.edu.pl

**Keywords:** scale model, small reverberation room, similarity criteria, transmission loss

## Abstract

This paper contains a detailed description of the design and validation of a measurement stand for testing the airborne sound insulation of specimens made at a small scale. The stand is comprised of two coupled reverberation rooms in which the geometry represents the full-size reverberation rooms used at the AGH University of Science and Technology at a 1:8 scale. The paper proves that both the scaled measurement stand and the testing methodology conform to the ISO 10140 standards, and that the obtained measurement uncertainty does not exceed the maximum values specified in ISO 12999-1. Moreover, the calculated uncertainty of measurements obtained for the 1:8 scale stand is comparable with the typical uncertainty given in ISO 12999-1 and the uncertainty obtained on the full-scale measurement stand. In connection with the above, the authors have proved that by using the scaled-down measurement stands, one can obtain reliable and repeatable results of measurements of airborne sound insulation.

## 1. Introduction

Tests using scaled-down models allow the significant reduction of the costs of the analysis of selected processes and phenomena occurring in nature. Moreover, in many cases, they allow experiments to be conducted which would be impossible at the full scale. For example, wind engineering issues concerning buildings of complicated geometry require model experiments performed in wind tunnels [1]. Physical scaled-down models are also used during the design of hydrotechnical facilities as a necessary supplementary component of the design process [2]. Analysis of literature on the subject matter indicates that despite the high potential of computer modelling, tests with scaled-down models are also used in acoustics. Their main advantage is the possibility for a very accurate representation of wave phenomena, mostly with regard to the diffraction and interference of acoustic waves [3,4,5]. For this reason, scaled-down tests are used in such areas as environmental, architectural, and building acoustics.

Model research conducted in the context of noise analysis in the urban environment are mostly related to sound propagation in space [6], sound screening by partitions [7,8], and the impact of façade types on sound propagation in densely developed areas [9,10]. Their undisputed advantage is the fact that scaling of the urban infrastructure items allows acoustic measurements to be made under strictly controlled conditions, which is impossible at the full scale. In the area of architectural acoustics, scaled-down models are mostly used for testing the sound absorption of spatial structures in interiors with qualified acoustics [11,12] and in the analysis of the relationship between the architectural solutions used in a room and its acoustic parameters [13,14,15,16]. In building acoustics, model tests are most often used to analyse the sound insulation properties of materials and acoustic structures. Full-scale measurements of sound insulation require very extensive measurement stands or in-situ measurements. This entails the use of a significant amount of materials, generates high costs, and can cause problems during the transport and storage of measurement specimens. Tests on a scaled-down stand limit or even eliminate these problems.

The literature review indicates two different approaches to model testing of sound insulation. The first approach involves testing real specimens in a stand with reduced linear dimensions and at a limited frequency. The other approach is related to tests during which the scaling of the entire system is maintained, specifically the geometry of the measurement rooms, the range of the analysed frequencies, and the geometry of the test specimens.

Examples of sound insulation tests of real specimens with the use of small coupled twin reverberation rooms are presented in the literature [17]. In line with the volume of each room (equal to 1.36 m^3^), the measurement range had a lower limit of 400 Hz. Paper [18] presents a measurement stand consisting of a coupled small reverberation room and an anechoic room. Such a stand enabled measurements of the absorption coefficient (α), transmission loss (TL), and insertion loss (IL) of the real specimens. Being below 2 m^3^, the volumes of the rooms limited the measurement range to 570 Hz and higher. The next example of a scaled-down measurement stand is a 0.8 m^3^ mobile reverberation room which, when inserted into a full-scale anechoic room, allowed the TL measurements of real specimens with the sound intensity method in the frequency range of 400 Hz and upwards [19].

The solution to the problem of having a lower limit to the measurement range tests on reduced measurement stands is to scale down the whole experiment—this entails the scaling of the specimen and the measurement frequencies. The model maintains the same original proportions between the acoustic wavelength and the linear dimensions of the test room and specimens. A test stand meeting these criteria was developed in the Building Research Institute (ITB) in Warsaw [20]. The models of two reverberation rooms were made at a scale of 1:5 to represent real rooms in the ITB laboratory, and to maintain linear dimensions, shape, and mutual location (the source room was placed above the receiving room). The volumes of the source and receiving rooms were 0.55 m^3^ and 0.46 m^3^, respectively, and the measurement window dimensions for testing the floor slab and wall specimens were 0.52 × 0.86 m (at a 1:1 scale, this equates to 2.6 × 4.3 m). The measurement stand was used for testing sound insulation from both airborne and impact sounds. The preliminary tests of homogenous specimens indicated a satisfactory conformity of airborne sound insulation characteristics between the specimens at the 1:5 and 1:1 scales. However, such a conformity was not obtained for the impact sounds. A similar stand is presented in the literature [21,22], although this is used for single type sound insulation measurements. The first paper includes a description of a stand for testing airborne sound insulation at the 1:10 scale. The stand consisted of two cuboid-shaped coupled rooms with volumes of 0.06 m^3^ and 0.05 m^3^ (at a 1:1 scale, this equates to 60 m^3^ and 50 m^3^, respectively). The measurement window dimensions were 0.30 × 0.40 m (3 × 4 m at a 1:1 scale). The stand was used to conduct pilot tests of the impact of the specimen fastening method (boundary conditions) on the obtained values of sound insulation. The stand described in the literature [22] was also made at a scale of 1:10 and was used to conduct model tests of impact sound insulation. The stand was a 0.9 m^3^ cuboid receiving room (at a 1:1 scale, this equates to 90 m^3^). However, the paper does not present the test results obtained on this stand.

The references mentioned above indicate that scaled tests of sound insulation are being undertaken. However, due to the absence of precisely defined criteria of similarity between the real system and the scaled model, the tests are conducted only for the simplest partition models—for homogenous specimens, usually omitting the impact of boundary conditions on the test results. Unfortunately, the reliability of the presented results can be questioned because the authors do not indicate whether their measurement stands and procedures meet the requirements for laboratory stands described in ISO 10140 [23,24]. Failure to meet the above requirements may influence the reliability, repeatability, and reproducibility of the measurement results. As a result, while transferring the observation results from the model to the real conditions, it is difficult to determine what influences the final value of the sound insulation of the real specimen: the scaled-down test methodology or the scaling of the specimen and the obtained insulation results relating to it.

The purpose of the research presented in this paper was to build a scaled measurement stand for testing the airborne sound insulation of scaled specimens that meets all standard procedures dedicated to full-size laboratory stands. In contrast to the scaled measurement stands presented so far in the literature, our stand is to be fully compliant with the ISO 10140 guidelines and the uncertainty of obtained results will not exceed the maximum values specified in ISO 12999-1. This ensures the reliable and repeatable measurement results of airborne sound insulation performed on the studied stand. It is planned to use the stand for further research in order to define the precise criteria of similarity between the scaled model and the real system for testing the sound insulation of various scaled specimens.

The paper presents a detailed description of the proposed measurement stand and its full validation procedure. First, the compliance of the stand with the ISO 10140 requirements is verified, i.e., issues related to the geometry and construction of the stand, elements of the measurement setup, and the characteristics of the acoustic field inside the reverberation chambers. Next, a comparative analysis of uncertainty of measurement results obtained on the tested scaled stand and its full-size equivalent is performed. The obtained uncertainty values are compared with the requirements of ISO 12999-1, which specifies the maximum and typical uncertainty values obtained during the tests of airborne sound insulation.

## 2. The Scaled Measurement Stand in the Context of ISO 10140 Requirements

**Figure 1:**
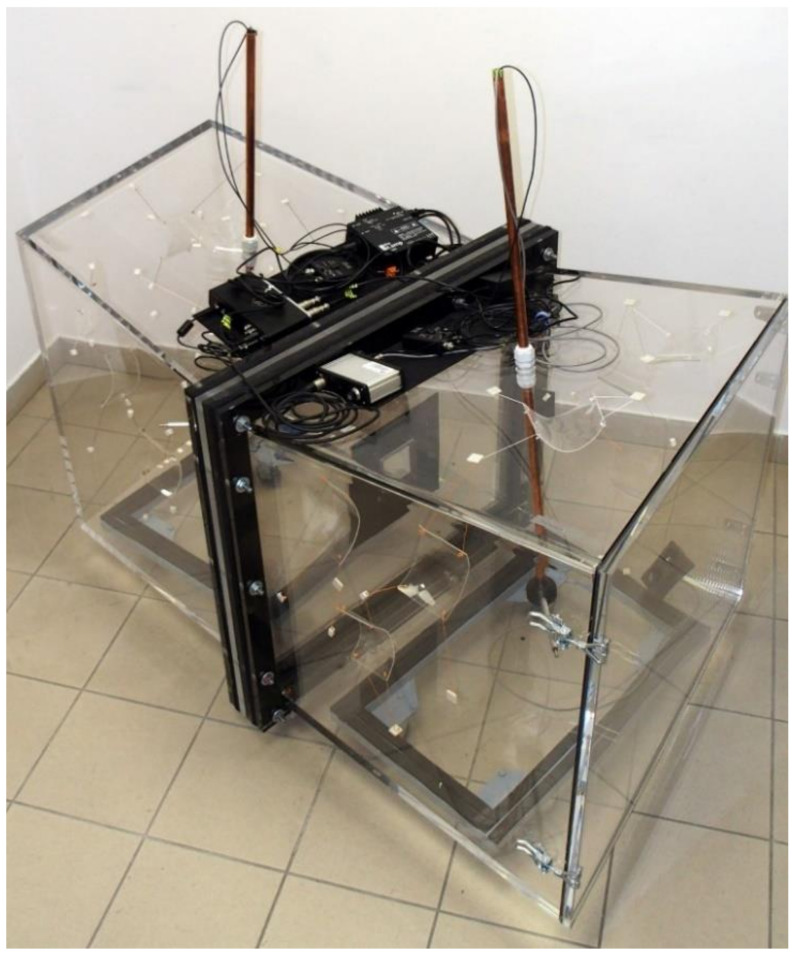
Coupled reverberation rooms in the 1:8 scale with equipment.

**Figure 2:**
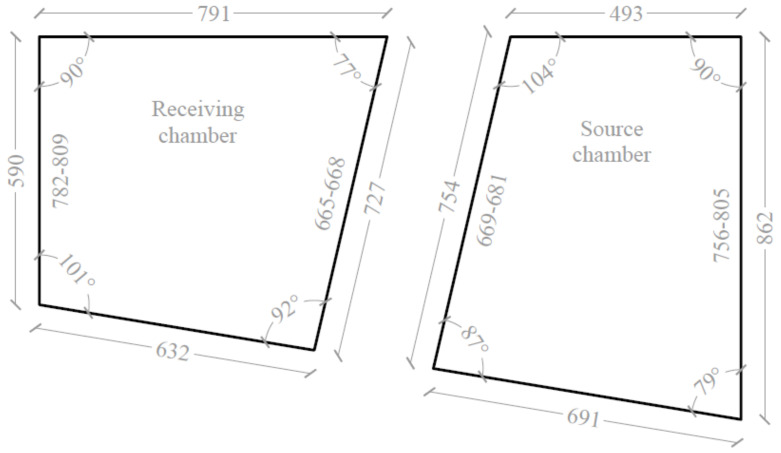
Detailed dimensions (in mm) of the coupled reverberation rooms at the 1:8 scale; the intervals define the walls heights that vary along the width.

The measurement stand presented in this paper consisted of two coupled reverberation rooms (Figure 1), the geometry of which directly represents the full-size reverberation rooms used at the Department of Mechanics and Vibroacoustics of AGH University of Science and Technology at a scale of 1:8. The volumes of the source and receiving rooms were about 0.35 m^3^ (at 1:1 scale, the volume of the rooms is almost 180 m^3^). No opposite walls in the rooms were parallel, the side walls were perpendicular to the floors. The detailed dimensions of the scaled source and receiving rooms are shown in Figure 2.

The walls of the scaled reverberation rooms were made of 20 mm-thick plexiglass panels. Two factors influenced the choice of this material. Firstly, the walls had to be transparent to facilitate the taking of both measurements and photographs. Secondly, the material had to be durable and resistant to possible mechanical damage. The wall thickness was chosen to ensure high sound insulation and minimise the transmission of sound by air. Each reverberation room was a separate structural component and was placed on a vibro-isolating mat on a mobile base. In the rooms, there was an opening on one side instead of the wall; this had a flange around the circumference with holes for bolts connecting the rooms. Before connecting the rooms, a double insert made of two 20-mm-thick plexiglass panels was placed between the flanges. A measurement window was made in the insert. The window had the dimensions as a specimen on the receiving room side, and it was slightly larger on the source room side to allow the mounting of the specimens. The whole system of coupled rooms was connected with 14 bolts. The rooms were designed specifically for the elimination of the lateral transmission of material sounds between rooms. Vibro-isolating mats with thicknesses of 2 and 15 mm were placed between the two parts of the insert and between the rooms’ flanges, respectively. In addition, the bolt holes in the flanges had rubber sleeves and double washers (steel and rubber) were used under the nuts. To allow access to the measurement window and the specimen mounting after the joining of the rooms, there was a door with a circumferential gasket and clamps in the source room wall opposite the measurement window. The door was made in the source room for technical reasons of mounting the specimens because the measurement window had larger dimensions on the source room side.

The tests in full-size rooms could be conducted in one of the two measurement windows: 1 × 2 m or 0.7 × 0.7 m. The 1:8 scale measurement stand offered six different measurement windows, each with a separate pair of inserts. The two basic measurement windows were a representation of the full-size room windows at the 1:8 scale, i.e., 125 × 250 mm (1 × 2 m at the 1:1 scale) and 87.5 × 87.5 mm (0.7 × 0.7 m at the 1:1 scale). In addition, inserts were prepared with measurement windows of the following dimensions: 250 × 500 mm (1 × 2 m full-size window at the 1:4 scale), 175 × 175 mm (0.7 × 0.7 m full-size window at the 1:4 scale), 350 × 350 mm (0.7 × 0.7 m full-size window at the 1:2 scale), and 676 × 615 mm (the largest possible measurement window between the scaled rooms).

**Figure 3:**
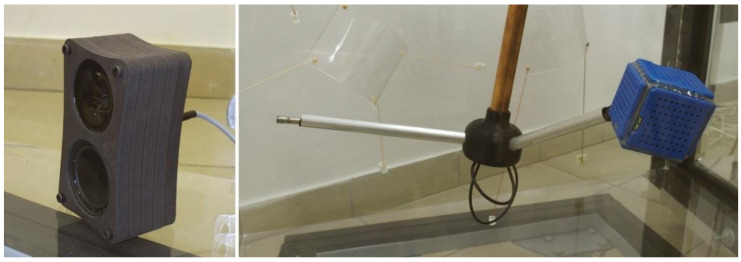
High-frequency sound sources: Architected Sound XS in the source room (**left**) and Architected Sound Omni Blue in the receiving room (**right**).

The measurement rig, which enabled the measurement of the airborne sound insulation, consisted of the following components. The measurement signals were generated by the specially designed and created high-frequency sound sources. The Architected Sound XS source (Figure 3, on the left) is a two-way set based on a 2″ dynamic transducer and a piezoelectric transducer. Its dimensions are 70 × 50 × 120 mm. The source can generate measurement signals in the 400 Hz–40 kHz frequency range. In the tests, it was used to generate the signal in the form of noise which was used to measure the sound insulation of a specimen. The maximum total sound power of the source is 97 dB. During the measurements, it was placed alternately in two bottom corners of the source room, opposite the measurement window. The receiving room had an Architected Sound Omni Blue omnidirectional sound source (Figure 3, on the right). It is built of six piezoelectric transducers and its dimensions are 53 × 53 × 53 mm. The source can generate measurement signals in the 400 Hz–80 kHz frequency range. In the tests, it was used to generate an MLS or swept-sine signal used to measure the reverberation time in the receiving room. The source was located at the end of a 12 mm-diameter and 150 mm-long aluminium tube connected with an 18 mm-diameter copper tube by means of a 3D-printed connector. The copper tube extended outside the receiving room through an opening sealed on both sides with a tight-fitting hydraulic gland with a nut on the room outside to inhibit the tube movement. Such a mounting of the source allowed its position to be controlled in all directions. The measurement data acquisition system comprised of two 1/4″ 46BE microphone sets from G.R.A.S of the first accuracy class, one in the source room, the other in the receiving room. The measurement range of the sets was 4 Hz–80 kHz, the dynamics range was 35 dBA–160 dB, and the sensitivity was 4mV/Pa. The method of mounting of the microphones was identical as the mounting of the speaker in the receiving room (see Figure 3, on the right). This ensured conformity with the ISO 10140-4 standard with regard to the minimum distance between the microphones and between the microphones and the room walls (min. 0.7 m–at the 1:8 scale this equates to just under 9 cm) and between the specimen and the sound source (min. 1 m, at the 1:8 scale this equates to just under 13 cm). The power to the microphone sets was supplied by 12AL modules from G.R.A.S. All measurement stand components were connected to a UMC204HD BEHRINGER U-PHORIA measurement card, which was in turn connected to the measurement computer.

**Figure 4:**
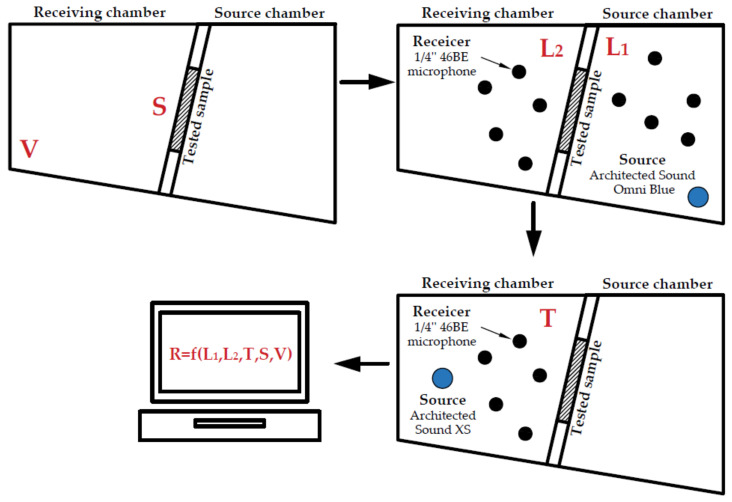
The flowchart of measurement methodology—subsequent stages of measurements and calculations of the parameters marked in red: sound pressure level in the source room ($L_{1}$) and in the receiving room ($L_{2}$), reverberation time in the receiving room (T), specimen surface area (S), receiving room volume (V), and airborne sound insulation of a specimen (R).

A dedicated computer programme was written for management of the measurements and analysis of results. The program was implemented in the MATLAB environment with the use of the ITA toolbox. The basic program modules include a module for reverberation time measurement based on the recorded impulse response and a module for the specimen sound insulation measurement based on the sound pressure levels recorded in the source and receiving rooms. The software enabled management of the scale of measurements, calibration of the reception devices, and verification of the measurement results on an ongoing basis. Figure 4 summarises the measurement methodology in the form of a flowchart.

**Figure 5:**
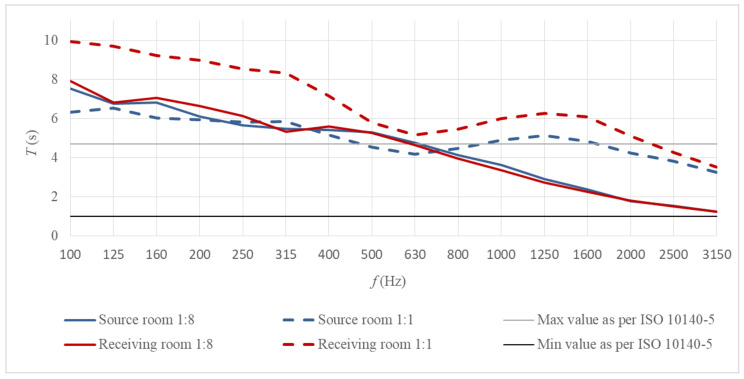
Reverberation times in the source and receiving rooms of the full-scale measurement stand and its counterpart at the 1:8 scale; the results from the scaled rooms were scaled to actual measurement frequencies.

In order to be used for laboratory tests of airborne sound insulation, the measurement stand must meet the strict requirements of ISO 10140-5. Consequently, the 1:8 scale stand was subjected to verification and qualification in accordance with that standard. The fulfilment of the condition of the minimum room volume (50 m^3^, which equates to 0.10 m^3^ at the 1:8 scale) and the proportion between the wall dimensions results directly from the fact that small coupled rooms at the 1:8 scale were accurate copies of the full-size rooms which meet the ISO requirements. The condition for the minimum measurement window size (10 m^2^, which equates to 0.16 m^2^ at the 1:8 scale) was met by adding the largest 0.42 m^2^ window to the set of inserts with measurement windows. The next requirement relates to the diffusivity of the sound field inside the source and receiving rooms. The ISO 10140-5 recommends the installation of diffusing elements in such a way that the measurement result is not influenced when further diffusing elements are installed. Based on this guideline, the sound diffusing elements were installed in both rooms. The elements were made of bent 2 mm-thick plexiglass panels. The dimensions, layout, and number of diffusing elements were tailored to the 1:8 scale stand according to the ISO 10140-5 guidelines; the diffusing elements were not scaled directly from the full-size stand [25]. The ISO 10140-5 standard also suggests that a too long or too short reverberation time in the reverberation rooms may disrupt the measurement results. If such a relationship is noticed, it is recommended to limit the reverberation time in the measurement bands from 100 Hz (at the 1:8 scale, this equates to 800 Hz) to the $1\;s\;–2{\left(V/50\;\right)}^{2/3}\;s$ range, which for coupled rooms gives 1.0–4.7 s. In the case of tests described in this paper, even though the suggested maximum reverberation time of 4.7 s was exceeded in the scaled-down rooms in the lowest frequency bands (see Figure 5), it was decided not to install additional sound absorbing elements. The scaled rooms were meant to represent the full-size rooms as accurately as possible, and the latter do not have sound absorbing elements although their reverberation time also exceeds the 4.7 s limit. However, during further work on the scaled stand, it is planned to design special sound absorbers and verify their impact on the results and uncertainty of measurements.

The next stage involved the determination of the minimum number and positions of sound sources in accordance with ISO 10140-5. All tested positions met the requirements of ISO 10140 5 in relation to the minimum distance between the source and partitions and between subsequent source positions; this is a minimum value of 0.7 m (at the 1:8 scale, this equates to just under 9 cm); however, at least two source positions must be a minimum of 1.4 m apart (at the 1:8 scale, this equates to 17.5 cm). The final choice was the minimum number of sound source positions according to ISO 10140-4 and ISO 10140-5, which is two positions. Both positions were close to the bottom corners of the source room opposite the measurement window, thus representing comparable positions of sound sources in the full-scale room.

This measurement stand was tested for the reliability of the obtained measurement results (comparison of measurement uncertainty on the 1:8 and 1:1 scale stands) and repeatability of results (based on the ISO 12999-1 requirements [26]).

## 3. Uncertainty of Measurements—Comparative Analysis of the Tested Stands

The verification of the reliability of the results obtained during the tests of sound insulation on the scaled measurement stand involved a comparative analysis of the uncertainty of measurement results obtained on the scaled stand and in the full-size rooms conforming to ISO 10140. The statistical analyses were made in the R environment using the following packages: readxl, knitr, vcdExtra, sjstats, dplyr, fame, stats, matrixStats, Alfapart, car, ggpubr, and lawstat. The obtained measurement uncertainty values were compared with the requirements of ISO 12999-1, which specifies the maximum and typical uncertainty values obtained during the tests of airborne sound insulation.

The experiment took place in the following way. Both measurement stands, the full-size and at the 1:8 scale, were used to measure the sound insulation of three specimens. Specimens made of 15 mm-thick plexiglass panels (dimensions 1 × 2 m and 0.7 × 0.7 m) and a 1 mm-thick steel specimen (dimensions 0.7 × 0.7 m) were tested on the full-size stand. Both plexiglass specimens on the scaled stand had all three dimensions faithfully scaled, but the steel specimen thickness was left the same and only the remaining two dimensions were scaled. The airborne sound insulation of a specimen is a function of the following input parameters: sound pressure level in the source room ($L_{1}$) and in the receiving room ($L_{2}$), reverberation time in the receiving room (T), specimen surface area (S), and receiving room volume (V). It is calculated according to the following formula [24]:

(1) $$R=L_{1}-L_{2}+10\mathrm{lg}\frac{TS}{0.16V}$$

Parameters $L_{1}$, $L_{2}$, T, and consequently also R, are dependent on sound frequency f. The sound insulation measurements are mainly performed in one third octave bands in the frequency range from 100 to 3150 Hz.

In order to test the variability of the input parameters, measurements of levels $L_{1}$ and $L_{2}$ and also reverberation time in the receiving room T were taken at 70 random measurement points placed in the reverberation rooms according to the ISO 10140-4 guidelines. The variability of S and V was specified a priori on the basis of the resolution of the measuring device. The signal used in the measurements of $L_{1}$ and $L_{2}$ levels was white noise and the MLS signal was used in the reverberation time measurements. The sound pressure measurements in the rooms were performed with two different speaker positions—35 measurement points for each position. The average time of each measurement was 8 s. The acoustic background was also measured in the receiving room; however, due to compliance with the minimum distance of the background from the signals as recommended in ISO 10140-4, these data were not included in the final analyses.

The uncertainty of the sound insulation measurement R was determined using the Monte Carlo method [27,28]. This method allows capturing the asymmetry of the uncertainty interval even at very small deviations of uncertainty intervals from symmetric intervals [29]. This results in a very accurate comparison of uncertainties of measurements performed on the analysed test stands, i.e., the full-size stand and the 1:8 scale stand. The uncertainty determination procedure in the Monte Carlo method involves multiple draws of input parameters to the measurement equation (10^6^ draws were used [30]). The empirical distribution of the measured parameters is then determined on the basis of the variability of the obtained results, and this distribution is used to determine the measurement uncertainty in the form of percentile interval [$p_{2.5\%}$, $p_{97.5\%}$]. The values $L_{1}$, $L_{2}$, and T were sampled from the measurement results obtained in the experiment. However, in order to introduce to the measurement equation (1), the uncertainty related to the measurement of parameters S and V, it was necessary to determine the variability interval of those parameters from which the values were to be sampled. The considered variability intervals of parameters S and V were calculated as the B type uncertainty intervals, assuming measurement resolutions of $\delta_{1}=0.001$ m for the 1:8 scale stand and $\delta_{2}=0.01$ m for the full-size stand. For a specimen of dimensions k x m, the variability range of its surface area is determined as follows:

(2) $$S:\left[k\cdot m-\sqrt{{\left(\frac{k\cdot \delta_{i}}{\sqrt{12}}\right)}^{2}+{\left(\frac{m\cdot \delta_{i}}{\sqrt{12}}\right)}^{2}},\;k\cdot m-\sqrt{{\left(\frac{k\cdot \delta_{i}}{\sqrt{12}}\right)}^{2}+{\left(\frac{m\cdot \delta_{i}}{\sqrt{12}}\right)}^{2}}\right].$$

Due to an irregular shape of the receiving room, the variability interval of parameter V was determined for the least favourable variant of adding the uncertainty from each dimension:

(3) $$V:\;\left[V-\delta_{i}*\sqrt{3},V+\delta_{i}*\sqrt{3}\;\right].$$

**Figure 6:**
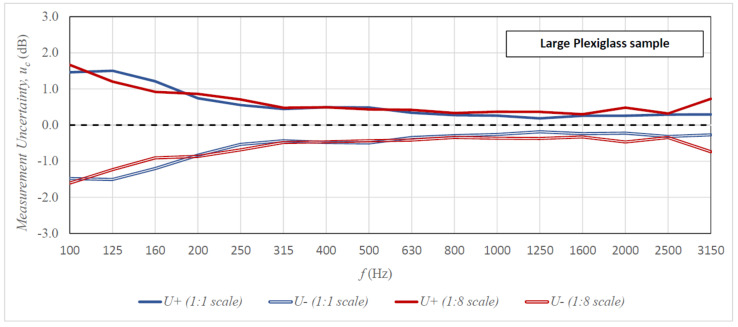
Uncertainty of sound insulation measurement for the 1 × 2 m plexiglass specimen (1:1 scale stand)/125 × 250 mm (1:8 scale stand) as a function of frequency for the 10-element measurement samples; the results from the scaled rooms were scaled to actual measurement frequencies.

**Figure 7:**
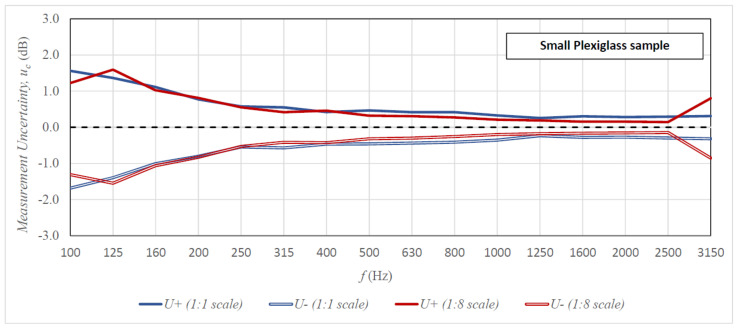
Uncertainty of sound insulation measurement for the 0.7 × 0.7 m plexiglass specimen (1:1 scale stand)/87.5 × 87.5 mm (1:8 scale stand) as a function of frequency for the 10-element measurement samples; the results from the scaled rooms were scaled to actual measurement frequencies.

**Figure 8:**
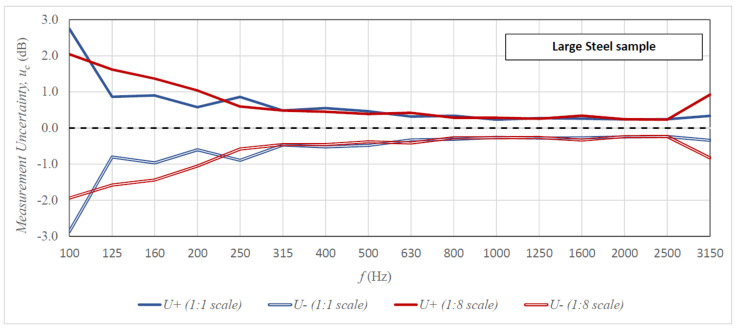
Uncertainty of sound insulation measurement for the 1 × 2 m steel plate specimen (1:1 scale stand)/125 × 250 mm (1:8 scale stand) as a function of frequency for the 10-element measurement samples; the results from the scaled rooms were scaled to actual measurement frequencies.

Figure 6, Figure 7 and Figure 8 present the uncertainty of the sound insulation measurements for all tested specimens as a function of frequency in the case of the 10-element measuring samples (comparable graphs for the 5-element and 15-element samples are included in Supplementary Materials). The graphs compare the results of tests on the 1:8 scale stand with the results obtained in the full-scale reverberation rooms. The results of the scaled tests were brought to the actual measuring ranges in accordance with the scale factor. The lower uncertainty of measurements is presented with a minus sign for better legibility. In addition, Table 1 includes a comparison of the greater uncertainty from the upper (*U+*) and lower (*U-*) measurement uncertainty with the maximum measurement uncertainty recommended by ISO 12999-1 and the typical value in the case of airborne sound insulation measurements [26] (comparable tables for the 5-element and 15 element samples are included in Supplementary Materials).

The analysis of results presented in Figure 6, Figure 7 and Figure 8 and in Table 1 allows the following conclusions to be drawn. The measurement uncertainty values obtained on both stands (full size and 1:8 scale) are similar over almost the entire range of measurement frequencies. There are, however, some discrepancies in the lowest bands of 100–200 Hz, but sometimes the uncertainty is greater on the full-size stand and sometimes on the scaled-down stand. The only frequency for which the measurement uncertainty is always greater on the 1:8 stand is 3150 Hz. This can be connected to the strong absorption of sound by air in this frequency range [31]; in reality, the measurement is performed for the frequency of 25.2 kHz, and only later was it scaled according to the scale factor. However, the uncertainty values at this frequency are so negligible that they do not significantly affect the reliability of the measurement result. The comparison of the obtained measurement uncertainties with the maximum standard deviations recommended by ISO 12999-1 indicates that in the case of measurement on the scaled-down stand, the maximum uncertainties were not exceeded in any frequency range. The results obtained on the full-size stand are similar, with one exception, the maximum uncertainty value in the 100 Hz band was exceeded for the 1 × 2 m steel specimen. The tested measurement stands also perform well for the typical uncertainty of results of airborne sound insulation measurement (see Table 1). In low frequency bands (100–200 Hz), the obtained uncertainties are close to the typical values or exceed them only slightly; in medium and high frequency ranges, they are lower than the typical values in most cases. The conclusion from these analyses is the statement that even for the minimum sample size [24] of 10 elements, we obtain satisfactory uncertainty of sound insulation measurements of specimens on the 1:8 scale stand. Table 2 presents an additional analysis of results, which indicates the exact size of the measurement sample at which the uncertainty of measurement results on all tested stands would be less than the maximum according to ISO 12999-1.

The presented statistical analyses were broadened to include an additional uncertainty budget in order to determine which uncertainty component made the greatest contribution to the final uncertainty of the measurement result. The contribution of individual uncertainty components was determined based on the uncertainty propagation law described by the formula:

(4) $$u_{c}\left(\hat{R}\right)=\sqrt{\overset{n}{\underset{i=1}{\sum }}c_{i}^{2}u_{i}^{2}},$$

where $u_{c}\left(\hat{R}\right)$ is the uncertainty of sound insulation measurement, $u_{i}$ is the uncertainty of individual input parameters to the measurement equation (partial uncertainty), and $c_{i}$ is the sensitivity factor determining the proportional contribution of a given input variable to the uncertainty result. Uncertainty $u_{i}=\{u_{L_{1}},\;u_{L_{2}},\;u_{T}\}$ of input parameters $L_{1}$, $L_{2}$, T was determined according to the following formula:

(5) $$u_{i}=\frac{s_{i}}{\sqrt{n}}t_{\left(n-1;0.975\right)},$$

where $s_{i}$ is the standard deviation of a given variable, *n* is the measurement sample size, and $t_{\left(n-1;0.975\right)}$ is the Student’s t-distribution quantile. Uncertainty of parameters S and V was determined based on the propagation of the B-type uncertainty, and the following uncertainty intervals were obtained for these parameters:

(6) $$u_{S}=\sqrt{{\left(\frac{k*\delta_{i}}{\sqrt{12}}\right)}^{2}+{\left(\frac{m*\delta_{i}}{\sqrt{12}}\right)}^{2}},$$

(7) $$u_{V}=\delta_{i}*\sqrt{3},$$

where k and m are the specimen’s dimensions, and $\delta_{i}$ is the measurement resolution (see Formulas (2) and (3)). Sensitivity factors $c_{i}$ of individual input parameters were calculated as partial derivatives of the measurement function with respect to a given input variable and the following values were obtained:

(8) $$c_{L_{1}}=c_{L_{2}}=1,$$

(9) $$c_{T}=\frac{10}{\ln\left(10\right)\bar{T}},$$

(10) $$c_{S}=\frac{10}{\ln\left(10\right)\bar{S}},$$

(11) $$c_{V}=\frac{10}{\ln\left(10\right)\bar{V}},$$

where $\bar{T}$, $\bar{S}$, and $\bar{V}$ are arithmetic means of the values from the measurement sample of parameters T, S, and V.

**Figure 9:**
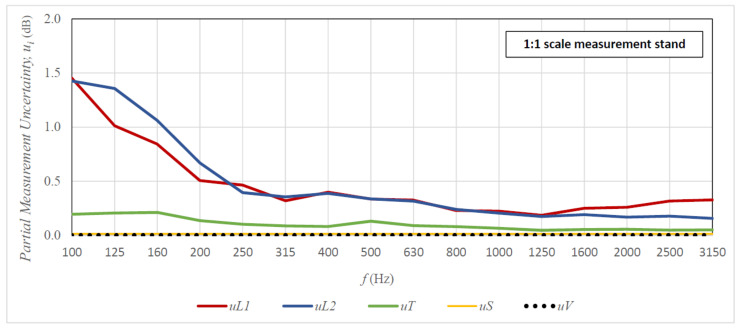
Share of measurement uncertainty of variables $L_{1}$ $L_{2}$, T, S, and V in the total uncertainty of sound insulation measurement of the 1 × 2 m plexiglass specimen; measurements on the full-size stand for the 10-element measurement sample.

**Figure 10:**
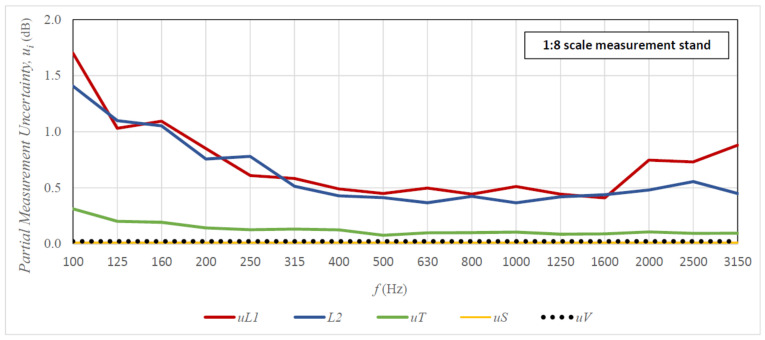
Share of measurement uncertainty of variables $L_{1}$, $L_{2}$, T, S, and V in the total uncertainty of sound insulation measurement of the 125 × 250 mm m plexiglass specimen; measurements on the 1:8 scale stand for the 10-element measurement sample. The results from the scaled rooms were scaled to actual measurement frequencies.

Figure 9 and Figure 10 present graphs of the share of partial measurement uncertainties (uncertainties of individual input parameters) in the total uncertainty of sound insulation measurement. The presented results are for the 1 × 2 m plexiglass specimen (full-size stand—Figure 9) and the 125 × 250 mm specimen (1:8 scale stand—Figure 10) for the 10-element measurement sample. The analysis of graphs shown in Figure 9 and Figure 10 indicates that the main components of the uncertainty of the sound insulation measurements on both tested stands are the uncertainties of input parameters $L_{1}$ and $L_{2}$. These dominate over the entire range of measurement frequencies and are significantly higher than the next significant partial uncertainty—the uncertainty of parameter T. The uncertainties of parameters S and V are negligibly low in comparison with the uncertainties of the other parameters. Moreover, the uncertainties of $L_{1}$ i $L_{2}$ are slightly higher in the scaled room than in the full-size room, while in the case of analysing the total uncertainty of sound insulation measurements, these differences are not that visible. The reason for this may be the correlations between signals in the source room and the receiving room, which are not considered in the case of partial uncertainties, which is in contrast to the testing of the total uncertainty using the Monte Carlo method. To summarise, in order to reduce the uncertainty of the obtained sound insulation results, the uncertainty of input parameters $L_{1}$ and $L_{2}$ should be reduced, e.g., by increasing the sample size.

Finally, one more analysis was conducted for verification of the possible use of a different measurement signal than the MLS to measure the reverberation time *T* in the scaled receiving room using an integrated impulse response method. The tested signal was Swept-Sine. The experiment involving the measurement of parameter T in 70 random measurement points in the receiving room was repeated, this time with both the MLS and the Swept–Sine signals. The obtained results were checked for the normality of their probability distribution. It was concluded that there were no reasons to reject the null hypothesis of normality of distributions in both cases. Consequently, it was possible to use the Levene’s test to compare the variances of the obtained results. The variance equality test indicated that for the 100, 200, 500, and 1000 Hz frequencies (measurement bands brought to the 1:1 scale), the obtained variances are different, and that for the three last frequencies, the result was at the significance limit (0.01<p<0.05). Smaller variances of results were obtained for the Swept–Sine signal, which suggests that it is a better signal for reverberation time measurement. However, the differences were small, and the share of the uncertainty of the reverberation time component in the total uncertainty of the result is secondary, so both tested measurements signals can be used in the measurements of sound insulation of the specimens.

## 4. Summary

The paper includes a detailed description of the scaled-down stand for measuring the airborne sound insulation using scaled-down specimens. The stand validation results were presented in the context of the reliability and repeatability of measurement results obtained on this stand. Firstly, it was proven that both the scaled measurement stand and the measurement methodology meet the requirements of ISO 10140 adapted to the scale factor. Secondly, it was concluded that the uncertainty of measurements on the tested stand meets the requirements of ISO 12999-1 for maximum uncertainty values. Moreover, the calculated uncertainties of results for the 1:8 scale stand are comparable with typical values given in ISO 12999-1 and with the uncertainties obtained on the full-size stand. These conclusions give grounds to consider the presented stand for measuring the sound insulation of scaled-down specimens as being adequate for further research. In addition, the analyses presented in the paper confirm that reliable and repeatable results can be obtained with the use of scaled-down measurement systems.

## Figures and Tables

**Table 1:** Uncertainty of sound insulation measurement for all tested specimens for the 10-element measurement samples vs. the ISO 12999-1 recommendations [26]; the results from the scaled rooms were scaled to actual measurement frequencies.

f (Hz)	ISO 12999-1 Recommendations	Greater of the Upper and Lower Uncertainties of Sound Insulation Measurement					
	Maximum Standard Deviation/Typical Standard Deviation	Measurements on the 1:8 Stand			Measurements on the 1:1 Stand		
		Plexiglass Specimen 125 × 250 mm	Plexiglass Specimen 87.5 × 87.5 mm	Steel Specimen 125 × 250 mm	Plexiglass Specimen 1 × 2 m	Plexiglass Specimen 0.7 × 0.7 m	Steel Specimen 1 × 2 m
100	2.6/1.4	1.50	1.31	2.04	1.48	1.68	2.87
125	2.2/1.2	1.71	1.59	1.62	1.51	1.39	0.86
160	1.9/1.0	1.50	1.06	1.44	1.21	1.11	0.97
200	1.7/0.9	0.93	0.83	1.05	0.83	0.80	0.60
250	1.5/0.8	0.60	0.55	0.59	0.55	0.58	0.90
315	1.4/0.7	0.55	0.42	0.49	0.45	0.57	0.49
400	1.3/0.6	0.58	0.46	0.46	0.49	0.46	0.55
500	1.3/0.6	0.39	0.32	0.39	0.49	0.46	0.48
630	1.3/0.6	0.35	0.31	0.42	0.35	0.44	0.33
800	1.3/0.6	0.32	0.27	0.28	0.29	0.42	0.34
1000	1.3/0.6	0.33	0.21	0.28	0.26	0.35	0.26
1250	1.3/0.6	0.37	0.19	0.26	0.19	0.26	0.28
1600	1.3/0.6	0.49	0.16	0.34	0.26	0.30	0.27
2000	1.3/0.6	0.63	0.16	0.24	0.26	0.29	0.25
2500	1.3/0.6	0.76	0.15	0.23	0.32	0.30	0.24
3150	1.3/0.6	0.73	0.85	0.92	0.30	0.32	0.34

**Table 2:** Minimum size of measurement sample that ensures keeping the uncertainty below the maximum value according to ISO 12999-1 [26]—values for the full-size stand and the scaled-down stand for all three tested specimens; the results from the scaled rooms were scaled to actual measurement frequencies.

f (Hz)	Minimum Size of Measurement Sample That Ensures Keeping the Uncertainty Below the Maximum Value According to ISO 12999-1					
	Measurements on the 1:8 Stand			Measurements on the 1:1 Stand		
	Plexiglass Specimen 125 × 250 mm	Plexiglass Specimen 87.5 × 87.5 mm	Steel Specimen 125 × 250 mm	Plexiglass Specimen 1 × 2 m	Plexiglass Specimen 0.7 × 0.7 m	Steel Specimen 1 × 2 m
100	4	3	7	4	5	13
125	4	7	6	5	5	3
160	3	4	6	5	5	3
200	3	3	4	3	3	3
250	3	3	3	3	3	4
315	3	3	3	3	3	3
400–2500	3	3	3	3	3	3
3150	4	5	7	3	3	3

## Data Availability

All data are include in paper or supplementary materials.

## Supplementary Materials

The following are available online at https://www.mdpi.com/article/10.3390/s21196663/s1: Figure S1—Uncertainty of sound insulation measurement for the 1 × 2 m plexiglass specimen (1:1 scale stand)/125 × 250 mm (1:8 scale stand) as a function of frequency for the 5 element measurement samples; Figure S2—Uncertainty of sound insulation measurement for the 0.7 × 0.7 m plexiglass specimen (1:1 scale stand)/87.5 × 87.5 mm (1:8 scale stand) as a function of frequency for the 5-element measurement samples; Figure S3—Uncertainty of sound insulation measurement for the 1 × 2 m steel plate specimen (1:1 scale stand)/125 × 250 mm (1:8 scale stand) as a function of frequency for the 5-element measurement samples; Figure S4—Uncertainty of sound insulation measurement for the 1 × 2 m plexiglass specimen (1:1 scale stand)/125 × 250 mm (1:8 scale stand) as a function of frequency for the 15 element measurement samples; Figure S5—Uncertainty of sound insulation measurement for the 0.7 × 0.7 m plexiglass specimen (1:1 scale stand)/87.5 × 87.5 mm (1:8 scale stand) as a function of frequency for the 15-element measurement samples; Figure S6—Uncertainty of sound insulation measurement for the 1 × 2 m steel plate specimen (1:1 scale stand)/125 × 250 mm (1:8 scale stand) as a function of frequency for the 15-element measurement samples; Table S1—Uncertainty of sound insulation measurement for all tested specimens for the 5 element measurement samples vs. the ISO 12999-1 recommendations; Table S2—Uncertainty of sound insulation measurement for all tested specimens for the 15 element measurement samples vs. the ISO 12999-1 recommendations.
