# Supplementary material for: Validation of a 1:8 Scale Measurement Stand for Testing Airborne Sound Insulation
Source: Sensors (Basel). 2021 Oct 7;21(19):6663. doi: 10.3390/s21196663 (PMC8512408; doi:10.3390/s21196663)
Supplement: Supplementary file 1 [file sensors-21-06663-s001.zip › sensors-1387525-supplementary.pdf]

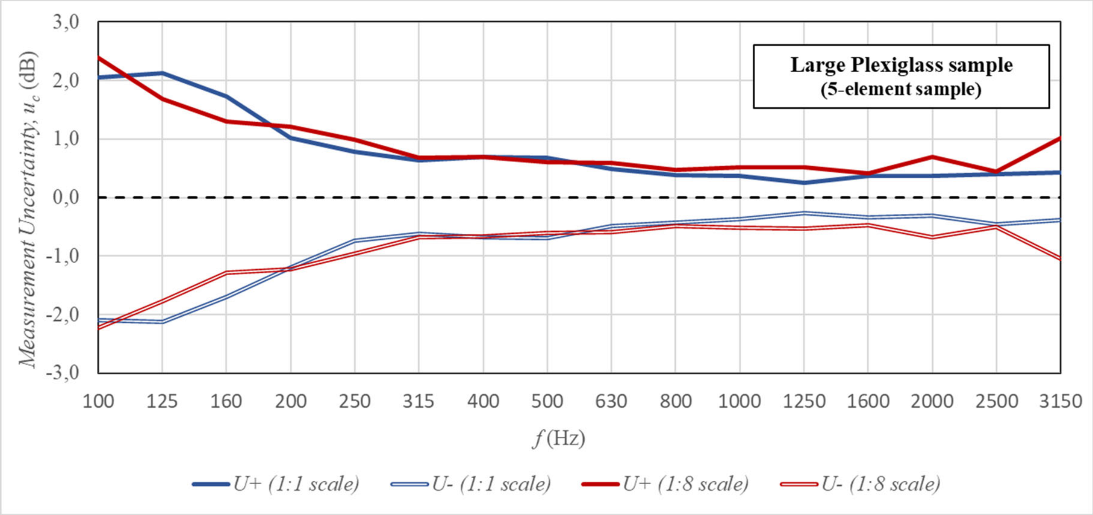

Figure S1. Uncertainty of sound insulation measurement for the 1 x 2 m plexiglass specimen (1:1 scale stand) / 125 x 250 mm (1:8 scale stand) as a function of frequency for the 5-element measurement samples. The results from the scaled rooms were scaled to actual measurement frequencies.

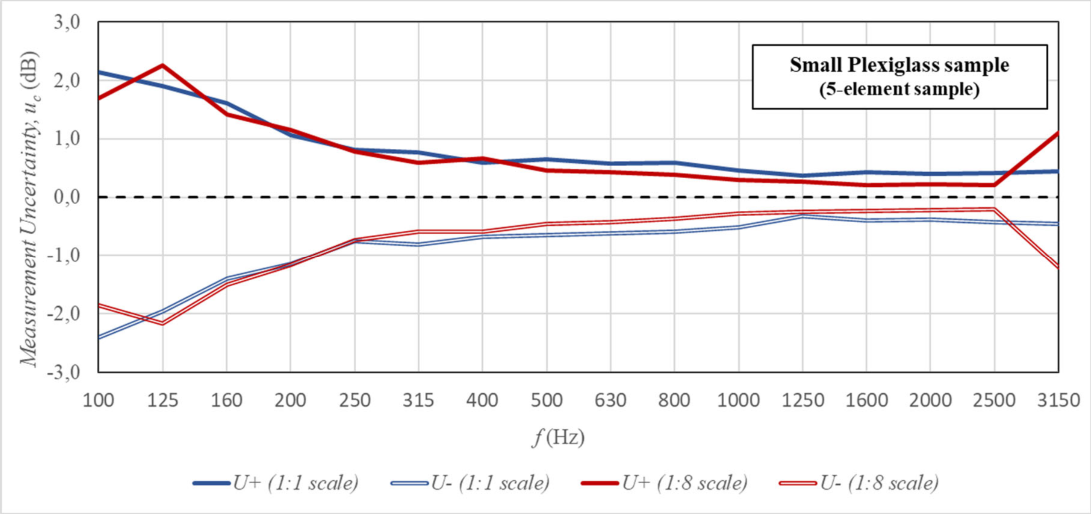

Figure S2. Uncertainty of sound insulation measurement for the 0.7 x 0.7 m plexiglass specimen (1:1 scale stand) / 87.5 x 87.5 mm (1:8 scale stand) as a function of frequency for the 5-element measurement samples. The results from the scaled rooms were scaled to actual measurement frequencies.

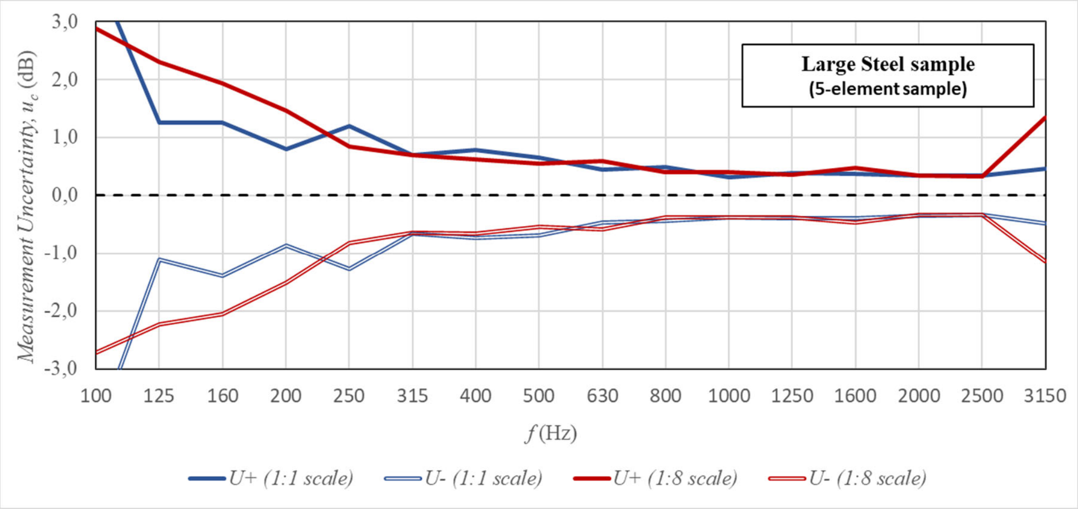

Figure S3. Uncertainty of sound insulation measurement for the 1 x 2 m steel plate specimen (1:1 scale stand) / 125 x 250 mm (1:8 scale stand) as a function of frequency for the 5-element measurement samples. The results from the scaled rooms were scaled to actual measurement frequencies.

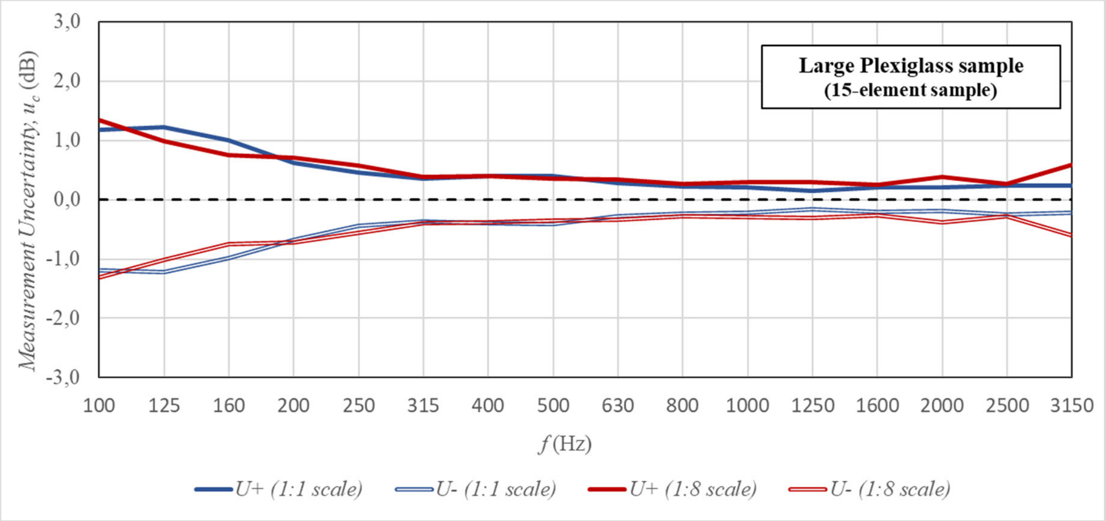

Figure S4. Uncertainty of sound insulation measurement for the 1 x 2 m plexiglass specimen (1:1 scale stand) / 125 x 250 mm (1:8 scale stand) as a function of frequency for the 15-element measurement samples. The results from the scaled rooms were scaled to actual measurement frequencies.

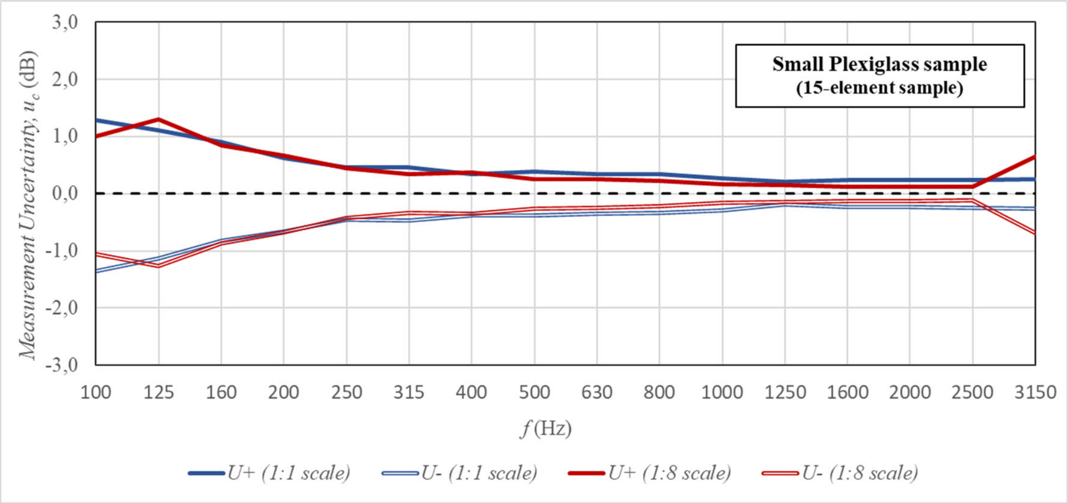

Figure S5. Uncertainty of sound insulation measurement for the 0.7 x 0.7 m plexiglass specimen (1:1 scale stand) / 87.5 x 87.5 mm (1:8 scale stand) as a function of frequency for the 15-element measurement samples. The results from the scaled rooms were scaled to actual measurement frequencies.

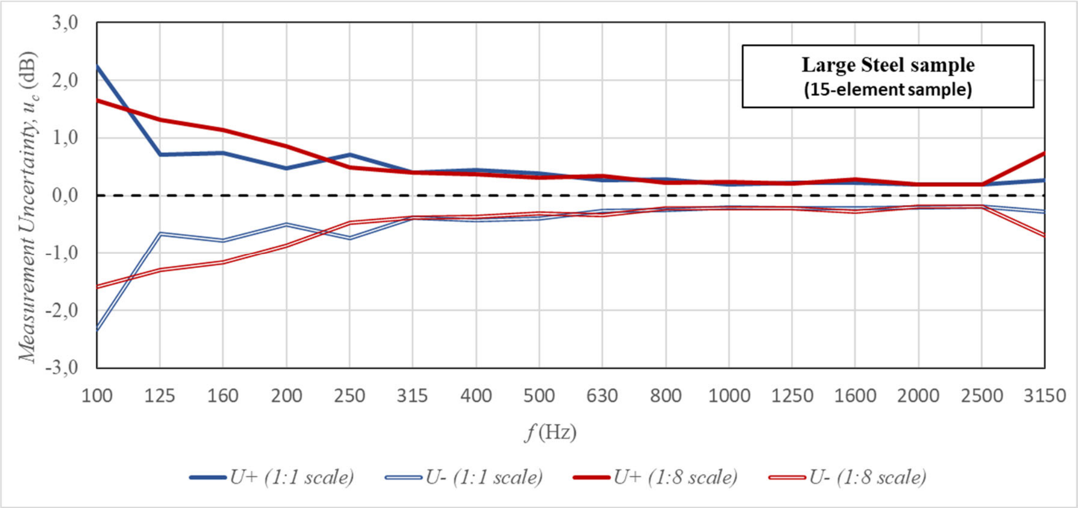

Figure S6. Uncertainty of sound insulation measurement for the 1 x 2 m steel plate specimen (1:1 scale stand) / 125 x 250 mm (1:8 scale stand) as a function of frequency for the 15-element measurement samples. The results from the scaled rooms were scaled to actual measurement frequencies.

Table S1. Uncertainty of sound insulation measurement for all tested specimens for the 5-element measurement samples vs the ISO 12999-1 recommendations [25]. The results from the scaled rooms were scaled to actual measurement frequencies.

f (Hz)	ISO 12999-1 recommendations	Greater of the upper and lower uncertainties of sound insulation measurement					
		Measurements on the 1:8 stand			Measurements on the 1:1 stand		
	Maximum standard deviation / Typical standard deviation	Plexiglass specimen 125 x 250 mm	Plexiglass specimen 87.5 x 87.5 mm	Plexiglass specimen 125 x 250 mm	Plexiglass specimen 87.5 x 87.5 mm	Plexiglass specimen 125 x 250 mm	Plexiglass specimen 87.5 x 87.5 mm
100	2.6 / 1.4	2.14	1.86	2.87	2.09	2.40	4.10
125	2.2 / 1.2	2.45	2.26	2.30	2.13	1.97	1.26
160	1.9 / 1.0	2.16	1.50	2.05	1.72	1.61	1.39
200	1.7 / 0.9	1.36	1.16	1.51	1.20	1.14	0.86
250	1.5 / 0.8	0.85	0.79	0.84	0.79	0.81	1.27
315	1.4 / 0.7	0.79	0.59	0.69	0.64	0.81	0.69
400	1.3 / 0.6	0.83	0.66	0.65	0.70	0.67	0.79
500	1.3 / 0.6	0.55	0.46	0.55	0.70	0.66	0.68
630	1.3 / 0.6	0.50	0.44	0.59	0.49	0.62	0.47
800	1.3 / 0.6	0.46	0.39	0.41	0.42	0.59	0.49
1000	1.3 / 0.6	0.47	0.30	0.40	0.37	0.51	0.38
1250	1.3 / 0.6	0.53	0.27	0.37	0.26	0.37	0.40
1600	1.3 / 0.6	0.74	0.23	0.48	0.37	0.44	0.39
2000	1.3 / 0.6	0.90	0.22	0.35	0.38	0.41	0.35
2500	1.3 / 0.6	1.09	0.20	0.33	0.45	0.42	0.34
3150	1.3 / 0.6	1.09	1.20	1.34	0.42	0.46	0.48

Table S2. Uncertainty of sound insulation measurement for all tested specimens for the 15-element measurement samples vs the ISO 12999-1 recommendations [25]. The results from the scaled rooms were scaled to actual measurement frequencies.

f (Hz)	ISO 12999-1 recommendations	Greater of the upper and lower uncertainties of sound insulation measurement					
		Measurements on the 1:8 stand			Measurements on the 1:8 stand		
	Maximum standard deviation / Typical standard deviation	Plexiglass specimen 125 x 250 mm	Plexiglass specimen 125 x 250 mm	Plexiglass specimen 125 x 250 mm	Plexiglass specimen 125 x 250 mm	Plexiglass specimen 125 x 250 mm	Plexiglass specimen 125 x 250 mm
100	2.6 / 1.4	1.22	1.06	1.66	1.19	1.35	2.32
125	2.2 / 1.2	1.40	1.30	1.31	1.23	1.13	0.71
160	1.9 / 1.0	1.22	0.86	1.16	1.00	0.90	0.79
200	1.7 / 0.9	0.76	0.67	0.86	0.67	0.66	0.50
250	1.5 / 0.8	0.49	0.45	0.48	0.46	0.47	0.73
315	1.4 / 0.7	0.45	0.34	0.39	0.36	0.47	0.40
400	1.3 / 0.6	0.47	0.37	0.37	0.40	0.38	0.44
500	1.3 / 0.6	0.32	0.26	0.31	0.40	0.38	0.39
630	1.3 / 0.6	0.29	0.25	0.34	0.28	0.36	0.27
800	1.3 / 0.6	0.27	0.22	0.23	0.24	0.34	0.27
1000	1.3 / 0.6	0.27	0.17	0.23	0.22	0.29	0.21
1250	1.3 / 0.6	0.30	0.15	0.21	0.15	0.21	0.23
1600	1.3 / 0.6	0.39	0.13	0.28	0.21	0.24	0.22
2000	1.3 / 0.6	0.51	0.13	0.20	0.21	0.23	0.20
2500	1.3 / 0.6	0.61	0.12	0.19	0.25	0.24	0.20
3150	1.3 / 0.6	0.58	0.69	0.74	0.24	0.26	0.28
